# Updates in the Management of Dengue Shock Syndrome: A Comprehensive Review

**DOI:** 10.7759/cureus.46713

**Published:** 2023-10-09

**Authors:** Rakshit K Singh, Aakriti Tiwari, Prasiddhi D Satone, Tannu Priya, Revat J Meshram

**Affiliations:** 1 Department of Paediatrics, Jawaharlal Nehru Medical College, Datta Meghe Institute of Higher Education and Research, Wardha, IND; 2 Department of Paediatrics, Pravara Institute of Medical Sciences, Shirdi, IND

**Keywords:** dengue shock syndrome, high-grade fever, vaccine, antiviral drugs, fluid resuscitation, thrombocytopenia, vascular leakage, cytokine

## Abstract

Dengue is a very serious public health problem that can manifest a wide range of symptoms from asymptomatic to fatal conditions, such as dengue shock syndrome (DSS). It is a life-threatening mosquito-borne viral infection widely spread in tropical areas. Dengue virus transmission occurs from an infected *Aedes* mosquito to humans. Various factors are responsible for the occurrence of the disease, such as viral load, age of the host, immune status of the host, and genetic variability. Dengue infection occurs in three phases: febrile, critical, and recovery. The febrile phase lasts for seven days and manifests symptoms such as high-grade fever, headache, arthralgia, and backache, and in some cases, the upper respiratory tract and gastrointestinal tract are also involved. Severe dengue is characterized by endothelial dysfunction that causes vascular permeability and plasma leakage. The fundamental mechanisms of these immune pathologies are not yet known. Dengue manifests various complications such as dengue encephalopathy, encephalitis, stroke, ocular involvement, acute transverse myelitis, myalgia, and cerebellar syndrome, but the most commonly seen is liver involvement. Dengue is managed supportively because there are no proven curative treatments. The cornerstone of care during the critical period of dengue is prudent fluid resuscitation. The first fluid of preference is a crystalloid. Prophylactic transfusion of platelets is not advised. The occurrence of four antigenically different dengue virus serotypes, each able to elicit a cross-reactive and disease-enhancing antibody response against the other three serotypes, has made the creation of the dengue vaccine a difficult undertaking. The development of a dengue vaccine has faced significant challenges due to a lack of the best animal models and a variety of immunological conditions in people, particularly in endemic locations. Dengvaxia is a live attenuated vaccine, which was developed by Sanofi. It is made up of four chimeric vaccine viruses produced by Vero cells.

## Introduction and background

Dengue fever has emerged as a persistent concern in the medical profession. The emergence of dengue shock syndrome (DSS), among its complex clinical manifestations, offers a difficult problem because of its medical illness that requires proper understanding, early diagnosis, prompt treatment, and a coordinated effort to lessen its effects [1]. The high prevalence of dengue sickness and frequent outbreaks put a significant strain on the nation's health services and economy. The three primary methods used in India to prevent and control the spread of the dengue virus are case detection, case management, and vector control [2]. Preventive approaches aim to lower the population density of the vectors, *Aedes aegypti* and *Aedes albopictus*, in the absence of a vaccine or particular antiviral treatment for dengue fever (DF) and dengue hemorrhagic fever (DHF). Insecticide use, public awareness campaigns, and surveillance for *Aedes aegypti* and *Aedes albopictus* larvae are the primary approaches to control DF/DHF in tropical countries. Due to the absence of a dedicated antiviral treatment for dengue, the development of an anti-dengue vaccination is a top priority. Inactivated virus vaccines, recombinant subunit vaccines, viral vector vaccines, DNA vaccines, and live attenuated virus vaccines are the five types of dengue vaccinations currently being developed [2]. Dengue fever is a crippling illness that causes an increased temperature, headache, joint pain, and skin rash; the development of DSS increases the severity of the disease to alarming levels, usually resulting in shock, hemorrhage, and organ dysfunction. DSS is an exciting but perplexing dilemma in infectious diseases because transitioning from a moderate dengue infection to the crucial point of DSS, it incorporates a sophisticated interaction combining viral kinetics, the body's immunological responses, and the resilience of blood vessels [3].

**Figure 1:**
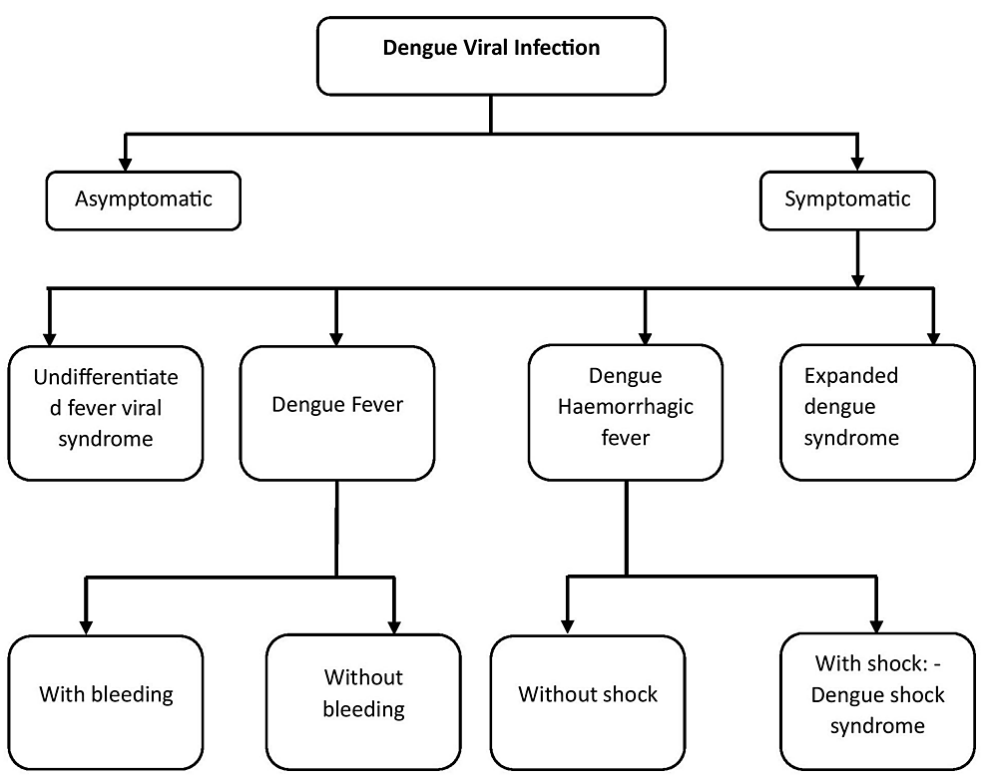
Clinical course of dengue fever Note: Author's creation

Clinical features of DSS include significant plasma leakage leading to shock, fluid accumulation in the pleural and abdominal regions, increased tendency for bleeding, and organ failure [4]. This intricate combination of immune responses, viral components, and blood vessel permeability transforms an initially risk-free viral infection into a dangerous condition. Despite advances in medical knowledge, exactly how DSS develops is still unclear. This highlights the need to understand better how the virus interacts with its host and the factors that promote the serious consequence [4]. There are various clinical courses of dengue infection, which is shown in Figure 1. Nowadays, there are various interventions or clinical trials that are ongoing to overcome the severity of dengue shock syndrome, and different kinds of interventions are explained in the later section.

## Review

Methodology

**Figure 2:**
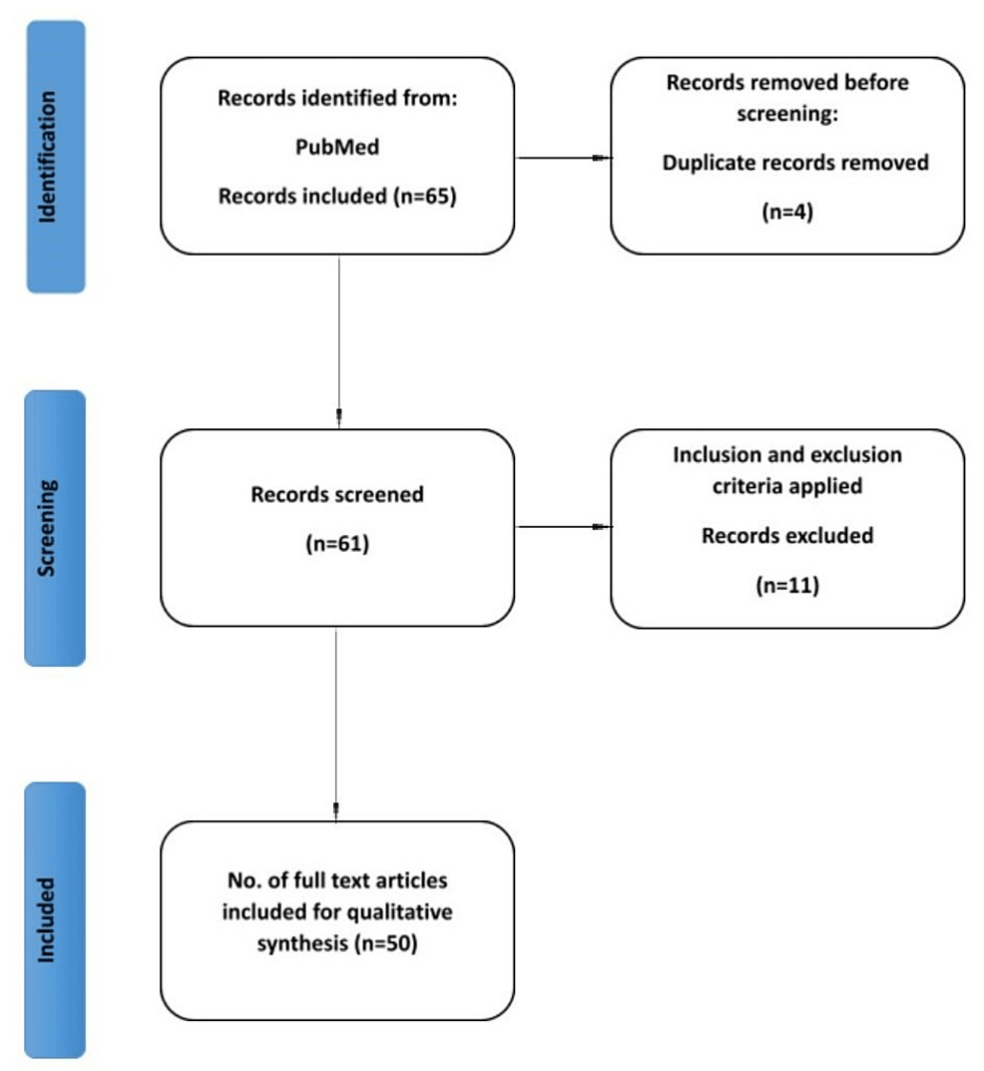
PRISMA flow diagram PRISMA: Preferred Reporting Items for Systematic Reviews and Meta-Analyses Note: Author's creation

An English literature search was undertaken using the internet databases PubMed and Google Scholar with the keywords "dengue shock syndrome," "fluid resuscitation," "thrombocytopenia," "vascular leakage," "cytokine," "high-grade fever," "vaccine," and relevant synonyms. The search covered papers published from the database inception to the present, with no explicit date constraints. This ensured that the most recent research on the subject until September 23, 2023, was included. Searching numerous databases, creating inclusion and exclusion criteria, screening papers, and choosing the final research for the review were all part of the procedure. Peer-reviewed articles published in English focusing on dengue shock syndrome are included. In contrast, paid articles, articles not in English, and articles not directly related to the topic are excluded. The initial screening consisted of reading the titles and abstracts of the identified papers by the inclusion and exclusion criteria. Full-text papers for possibly relevant research were retrieved, and further screening was performed to pick the final articles for the review. The inclusion criteria were satisfied by 49 papers included in the final review. The search methodology by the Preferred Reporting Items for Systematic Reviews and Meta-Analyses (PRISMA) method is shown in the flow diagram in Figure 2.

Pathophysiology

When the virus enters the body for the first time, it triggers a coordinated series of immune responses that starts off the journey from dengue infection to the start of DSS. Immune cells start a complicated interplay of cytokines and chemokines when the virus establishes itself, causing inflammation and triggering the immune system. This immune response can occasionally become too active, which can cause endothelial dysfunction and increase the permeability of blood vessels [5]; then, when fluid diffuses from blood arteries into adjacent tissues, hypovolemic shock or decreased blood volume results, which is the hallmark of DSS and the loss of plasma [5]. Clinical features of dengue shock syndrome differentiate the alarming signs and symptoms (Table 1).

**Table 1:** Clinical features of dengue shock syndrome differentiating the alarming signs and symptoms Note: Author's creation

Dengue without warning signs	Dengue with warning signs
Nausea	Abdominal pain or tenderness
Rash	Persistent vomiting
Headache	Ascitis, pleural effusion
Leukopenia	Mucosal bleeding
Vomiting	Hepatomegaly > 2 cm

The complexity of DSS extends beyond its clinical manifestation and includes a wide range of challenges. Due to the variety of clinical manifestations of DSS and the lack of specific biomarkers, timely diagnosis of this syndrome is slightly challenging [6]. As a result, medical professionals typically depend on clinical criteria and supporting test evidence to identify patients at risk for developing DSS. Rapid intervention is necessary because of DSS's quick progression since it frequently calls for close monitoring, fluid replacement, and specialized treatment strategies [6]. Clinical judgment and a thorough understanding of the developing pathophysiology are required to achieve the delicate balance between giving fluids to prevent shock and avoiding fluid excess. This review investigates the complex aspects of dengue shock syndrome and also aims to sort out the complexities, challenges, and potential solutions within this significant clinical occurrence. Our goal is to shed light on how to better understand, identify, and manage DSS by thoroughly assessing its global relevance, immune system responses, probable consequences, and management techniques. Our goal is to give healthcare professionals and researchers the fundamental information necessary to negotiate this challenging environment and, in the end, lessen the effect of this deadly infectious threat.

Immunopathology

The interplay between viral dynamics and host immune responses gives rise to the complex immunopathological cascade that defines DSS, inviting exploration into the intricate mechanisms that underpin its evolution. The dengue virus enters the host's bloodstream through an infected *Aedes* mosquito, infiltrating immune cells and triggering events that stimulate both protective and destructive responses. The initial encounter between the virus and dendritic cells initiates a cascade and stimulates the production of cytokines and interferon. In response to the pro-inflammatory environment, immune cells mobilize to fight the virus while unintentionally harming the host's tissues [7,5]. This chaotic war takes place inside the host. Antibody-dependent enhancement (ADE) plays a pivotal role in shaping the immune landscape, as pre-existing antibodies from a previous infection may facilitate, rather than impede, viral entry into target cells [8]. This paradoxical enhancement of viral uptake can lead to heightened viral replication, intensifying the inflammatory response and ultimately contributing to the pathogenesis of DSS [9].

Endothelial cells, lining the interior of blood vessels, emerge as pivotal players in the immunopathological narrative of DSS. In the face of viral invasion and the ensuing immune assault, these sentinel cells witness a cascade of events that threaten vascular integrity [10]. Cytokines released in response to infection elicit endothelial activation characterized by increased expression of adhesion molecules. This heightened adhesiveness facilitates the infiltration of immune cells into the vascular wall, contributing to vascular leakage and plasma extravasation [11]. Plasma leakage leading to hypovolemia and shock are the characteristic symptoms of DSS caused by the resultant imbalance in fluid dynamics [12]. Tumor necrosis factor-alpha (TNF-alpha), interleukin-6 (IL-6), and interleukin-8 are among the pro-inflammatory cytokines that play an essential role in the immune responses that control the course of the diseased host [13]. However, in the case of overproduction of these cytokines, it will lead to abnormal endothelial activation and vascular permeability [12]. This cytokine storm occurs due to various factors, i.e., viral load, host genetics, and immune history of the host. The hemorrhagic symptoms that characterize severe dengue infections are caused by the interaction of endothelial dysfunction, pro-inflammatory cytokines, and aberrant coagulation [14].

The complexity of DSS necessitates an integrated strategy that includes early detection, monitoring, and individual treatment plans. Fluid resuscitation, a crucial component of DSS therapy, must be balanced with sensitive fluid dynamics to restore intravascular volume without causing fluid overload [15]. Antiviral drugs and immunomodulatory therapy are two pharmacological interventions used to control the immune system and lessen the severity of illness [16].

Complications

The effect of DSS ripples across the cardiovascular, hematologic, hepatic, and neurological systems, presenting a series of problems that require comprehensive care [17,18]. Disruption of vascular integrity, which results in plasma leakage as its defining characteristic, is one of the significant consequences of dengue shock syndrome [17]. The interaction of viral invasion, immunological response, and endothelial stimulation leads to this vascular instability. Endothelial cells are activated, and their expression of adhesion molecules is raised due to cytokines generated in response to infection [19]. This increased adhesiveness enables immune cells to enter the arterial wall, increasing fluid extravasation and vascular permeability [20].

The most common complication of dengue shock syndrome is the involvement of the liver, with significant implications for disease progression [21]. Hepatocytes, the primary functional cells of the liver, bear the brunt of the viral assault, leading to hepatocellular injury and the release of liver enzymes. Elevated transaminase levels are a hallmark of hepatic dysfunction in DSS, reflecting the ongoing battle between immune responses and viral replication [22,23]. The intricate relationship between the dengue virus and the hematologic system further compounds the complexity of DSS. Thrombocytopenia, characterized by decreased platelet count, is a consistent feature of severe dengue infections, including DSS [24]. The resulting thrombocytopenia contributes to the bleeding diathesis observed in DSS, as a reduced platelet count compromises hemostasis and predisposes individuals to hemorrhagic manifestations [25].

Neurological complications cast a shadow of concern over DSS, albeit less frequently observed than other manifestations. The dengue virus invades various tissues and can sometimes infiltrate the central nervous system (CNS), giving rise to neurological sequelae [26]. Encephalopathy, characterized by altered mental status and neurological dysfunction, can manifest as a direct result of viral invasion or secondary to the systemic effects of severe infection [27]. Seizures, although less common, can further complicate the clinical picture [28,29].

Management

The management of DSS complications transcends symptom alleviation; it demands a holistic strategy addressing the underlying pathophysiology and systemic impact [30]. Fluid management, a cornerstone of DSS treatment, must be approached meticulously to restore intravascular volume without exacerbating plasma leakage [31]. Pharmacological interventions, such as vasopressors and blood product transfusions, aim to stabilize cardiovascular dynamics and counteract coagulopathies [32]. Early recognition of bleeding tendencies prompts vigilant measures to manage and correct coagulopathy, preventing the progression of hemorrhagic complications. In navigating the intricate complications of DSS, research efforts are pivotal to unraveling the underlying mechanisms and informing innovative interventions. Exploring immunomodulatory agents, such as corticosteroids and monoclonal antibodies, offers potential avenues to temper the exuberant immune response and mitigate disease severity [33]. Clinical trials exploring antiviral drugs and therapeutic interventions hold promise for directly targeting viral replication and influencing disease progression. Managing dengue shock syndrome (DSS) presents a multifaceted challenge that demands a nuanced approach, swift intervention, and a comprehensive understanding of the evolving clinical landscape. As a critical manifestation of dengue fever, DSS plunges individuals into a precarious state of shock, plasma leakage, and multi-organ dysfunction, necessitating a delicate balance between supportive care and targeted therapeutic strategies. This review delves into the intricate dimensions of DSS management, encompassing early recognition, fluid resuscitation, pharmacological interventions, and the promising horizon of emerging treatments.

*Early Recognition and Vigilant Monitoring*

The cornerstone of DSS management lies in early recognition and vigilant monitoring. Swift identification of warning signs, often guided by clinical criteria such as the WHO dengue classification, is crucial to initiate timely interventions. Particularly in endemic areas or during dengue epidemics, healthcare professionals must keep a high index of suspicion. Close observation of vital signs, fluid balance, laboratory results, and clinical condition enables early identification of imminent shock and directs treatment choices [12]. Hematocrit value, platelet count, liver enzyme, and coagulation profile monitoring regularly aid in understanding the changing pathophysiology of DSS and guide therapy modifications [34]. Normal hematocrit values as per age are shown in Table 2. To assess the level of plasma leakage and choose the best course of treatment, hematocrit monitoring is utilized. If a dengue patient's hematocrit value is consistently high with unstable vitals, i.e., metabolic acidosis, poor urine output, and tachycardia, this indicates active leakage of plasma and requires early fluid replacement therapy, and if the patient has low hematocrit value and unstable vitals, this indicates hemorrhage and requires blood transfusion [34].

**Table 2:** Normal hematocrit values as per age Note: Author's creation

Age	Hematocrit (%)
Neonates	44-65
Toddles (1-3 years old)	29-40
Child (4-10 years old)	31-43
Adult male	40-50
Adult female	36-46

Fluid Resuscitation: Striking the Delicate Balance

Fluid resuscitation is the most important and desirable for DSS management, aiming to restore intravascular volume, maintain organ perfusion, and counteract the effects of plasma leakage [35]. However, the difficult part is balancing the need for proper fluid replenishment with avoiding fluid excess. The prudent administration of crystalloids, colloids, and blood products must cater to each patient's specific needs and be supported by ongoing fluid status monitoring and dynamic fluid status evaluation [36]. The volume and amount of fluid infused can be directed by hemodynamic state (consciousness level, heart rate, peripheral pulse, blood pressure, capillary refill time, and urine output), as well as laboratory markers (hematocrit and platelet count). Treatment decisions are done in accordance with the 2009 WHO classification of patients based on their severity, warning symptoms, and concomitant illnesses, i.e., pregnancy, morbid obesity, diabetes mellitus, renal or cardiovascular impairment, hepatopathy, and hemolytic disorders [37]. In patients with warning signs and symptoms, crystalloid solution should be administered immediately at the rate of 10 mL/kg of body weight in the first hour. Re-evaluation should be done; if urine output is ≥1 mL/kg/hour and there is improvement in the patient's condition, the intravenous fluid rate can be reduced to 5-7 mL/kg/hour for 2-4 hours; if the patient's status allows, the fluid rate can be tapered to 3-5 mL/kg/hour for 2-4 more hours, and if improvement continues, the dose should be reduced to 2-4 mL/kg/hour; and if the patient condition deteriorate and hematocrit values rise rapidly, the rate should be increased to 5-10 mL/kg/hour for 1-2 hours. After three loads of 10 mL/kg/hour, if the condition does not improve, the case will be treated with shock as if it were severe dengue [37]. In patients with dengue shock, it is advised to start aggressive fluid resuscitation by infusing 20 mL of crystalloid fluid/kg of body weight over the course of 15-30 minutes. After that, the clinical situation should be re-evaluated to see if it has improved and if the patient's shock symptoms go away; fluid intake should be reduced to 10 mL/kg/hour for 1-2 hours while closely monitoring the patient's hemodynamics and hematocrit. If the clinical course is satisfactory, keep tapering the drip for another 4-6 hours at a rate of 5-7 mL/kg/hour. Tapering should be continued to maintain hydration at a rate of 2-4 mL/kg/hour for 24-48 hours [37]. Emerging medical technologies such as point-of-care ultrasonography and pulse contour analysis provide invaluable insights into real-time hemodynamic parameters, enabling accurate fluid titration.

Pharmacological Interventions: Navigating Complex Terrain

Pharmacological therapies become crucial in treating DSS in refractory shock or coagulopathies [16]. Vasopressors can improve vascular tone, maintain blood pressure, and restore tissue perfusion. Examples include dopamine and norepinephrine [38]. Vasopressor should be infused in the first hour when the fluid therapy is not sufficient to achieve the resuscitation target. These drugs are carefully titrated to strike a perfect balance between enhancing hemodynamics and reducing possible consequences. Platelets and fresh frozen plasma infusions treat coagulopathies and lessen bleeding tendencies [39]. Patients with dengue and the attending clinicians are frequently concerned about thrombocytopenia. Although platelets are routinely ordered in most hospitals, there are no clear recommendations for the therapy of thrombocytopenia. Prophylactic platelet transfusions are those that are provided before clinical bleeding occurs, as opposed to therapeutic platelet transfusions that are given to patients who are already bleeding [40]. Even at less than 20,000/cumm, prophylactic platelet administration is generally not recommended. In the absence of bleeding symptoms, a prophylactic platelet transfusion of less than 10,000/cumm may be administered [40]. The administration of blood products, guided by laboratory findings and clinical judgment, is pivotal in preventing and managing hemorrhagic complications. The most widely used vasopressor and their effects are shown in Table 3. The advantages of the early use of vasopressors include the following: norepinephrine administration could accelerate recovery from hypotension and thereafter stop persistent, severe hypotension; various pathways could result in an increase in cardiac output from norepinephrine infusion, and one of them is that norepinephrine might elevate cardiac preload and lessen dependence on preload; in cases of severe hypotension, early norepinephrine treatment may recruit microvessel growth and enhance microcirculation by raising organ perfusion pressure; and a dangerous fluid overload could be avoided with early infusion of norepinephrine. Positive fluid balance is independently linked to worse outcomes in septic shock, as is well known [41].

**Table 3:** Widely used vasopressor and their effects Note: Author's creation

Agents	Effects
Norepinephrine	Increases vascular tone and contractility
Epinephrine	Increases venous and arterial tone, contractility, and heart rate
Dopamine	Increases contractility and heart rate and increases renal and mesenteric vasodilation
Vasopressin	Increases vascular tone, platelet aggregation, and water retention

Immunomodulatory Interventions: A Promising Frontier

The exuberant immune response observed in DSS has prompted exploration into immunomodulatory interventions to temper the cytokine storm and mitigate disease severity. With their anti-inflammatory properties, corticosteroids have garnered attention as potential agents to modulate immune dysregulation [42]. Clinical trials investigating the role of corticosteroids in DSS management have yielded varied results, underscoring the need for further research to delineate their precise character, optimal dosing, and potential benefits. Monoclonal antibodies targeting specific cytokines or inflammatory pathways also hold promise as adjunctive therapies to attenuate the cytokine cascade and dampen immune-mediated vascular permeability [43].

Emerging Treatments and Future Directions

The management of dengue shock syndrome is continuously evolving and propelled by advancements in research and the pursuit of innovative treatments. Antiviral medications act directly on the dengue virus, reduce viral replication, and inhibit disease progression [44]. Targeted antiviral therapy is becoming more likely because of ongoing clinical trials examining the effectiveness and safety of antiviral drugs. A paradigm change in DSS management has also been brought by introducing dengue vaccines with curative and preventive action. These vaccines, which aim to provide immunity against all four dengue serotypes, can potentially lower the prevalence of severe illness and the effects of DSS [44]. A dedication to innovation and ongoing improvement characterizes the changing dengue vaccine research environment. Current clinical studies aim to improve the safety and efficacy of vaccines by modifying their formulations, dosage schedules, and methods. The various drugs currently under clinical trial for the treatment of dengue shock syndrome are mentioned in Table 4. To address current issues and maximize vaccination-induced immunity, adjuvants, immune-enhancing substances, and alternative vaccine platforms are being investigated.

**Table 4:** Drugs in phase 3 clinical trial for dengue shock syndrome NaCl: sodium chloride, 9vHPV: 9-valent human papillomavirus, TDV: tetravalent dengue vaccine Note: Author's creation

Intervention	Phase
Biological: live, attenuated, recombinant dengue serotype 1, 2, 3, and 4 viruses; biological: placebo: NaCl 0.9%	Phase 3
Biological: 9vHPV vaccine, biological: TDV	Phase 3
Drug: 2 days of ivermectin, drug: 3 days of ivermectin, drug: placebo	Phase 2/3
Drug: hypertonic sodium lactate, drug: Ringer’s lactate	Phase 3

Vaccine Strategies 

In the relentless battle against infectious diseases, vaccines have emerged as a formidable tool, heralding victories over once-debilitating conditions. Within this landscape, the quest to combat dengue fever, a mosquito-borne viral illness of global significance, has taken on renewed vigor, fueled by pursuing a comprehensive vaccine strategy to mitigate its most severe manifestation, dengue shock syndrome (DSS). Developing and deploying a dengue vaccine can transform the trajectory of DSS, alleviating its burden on individuals, communities, and healthcare systems [45].

The Path to a Dengue Vaccine: Navigating Serotype Diversity

The dengue virus exists as four distinct serotypes (DENV-1, DENV-2, DENV-3, and DENV-4), each capable of causing disease. The intricate dance between these serotypes and the human immune system has posed a unique challenge in vaccine development. Prior immunity to one serotype can paradoxically enhance disease severity upon subsequent infection with a different serotype, a phenomenon known as antibody-dependent enhancement (ADE) [9,46]. As such, a successful dengue vaccine must elicit a protective immune response against all four serotypes while avoiding the pitfalls of ADE.

Vaccine Approaches: From Live Attenuated to Tetravalent Formulations

The search for vaccines that can protect against dengue has led to the exploration of many vaccination platforms. Live attenuated vaccines, among them, have shown potential in simulating natural infection without illness. Tetravalent dengue vaccines, which include attenuated versions of all four serotypes, are a prominent contender in this category [47]. Exposing the immune system to antigens from each serotype promotes the development of neutral antibodies. Clinical trials of tetravalent vaccines have shown varying degrees of efficacy, with some protection against severe disease.

Challenges and Considerations: Safety, Efficacy, and Serotype Interactions

There are various difficulties in the process of creating a dengue vaccine. Safety and efficacy must always be balanced since any potential vaccination must produce a robust immune response without causing harmful side effects [48]. The intricacies of serotype interactions further complicate vaccine design, necessitating a delicate equilibrium between inducing protective immunity and avoiding ADE. The investigation of booster doses and alternate dosage regimens is prompted by the loss of vaccine-induced immunity over time, which adds another level of complication [9,48].

Integration Into Public Health Strategies: A Multipronged Approach

The successful implementation of a dengue vaccine strategy extends beyond the laboratory bench, requiring a multipronged approach that encompasses public health policies, vaccination campaigns, and community engagement. Dengue-endemic regions, characterized by high transmission rates, stand to benefit the most from a comprehensive vaccination program [49]. Targeted vaccination of high-risk populations, such as children and individuals with a history of dengue infection, can reduce disease and prevent severe outcomes such as dengue shock syndrome.

Realizing the Potential: Lessons From Dengue-Endemic Regions

Countries grappling with endemic dengue transmission have become testing grounds for dengue vaccine implementation [50]. Environmental elements such as stagnant water where mosquitoes breed, poor housing conditions, a lack of air conditioning, and meteorological conditions (i.e., temperature, precipitation, and humidity) increase the abundance, dispersion, and risk of exposure to *Aedes aegypti* in dengue-endemic locations [50]. Lessons learned from these areas offer perceptions on the prospects and obstacles of implementing a dengue vaccine into current healthcare systems.

## Conclusions

The complications of dengue shock syndrome epitomize the intricate dance between viral invasion, immune responses, and organ system dysfunction. From the hallmark feature of plasma leakage to the complexities of hepatic involvement, thrombocytopenia, and potential neurological sequelae, the implications of DSS cascade through multiple dimensions of health and demand a comprehensive approach to patient care. Developing a dengue vaccination approach is a flash of hope to lessen the effects of DSS and severe dengue infections. Creating, distributing, and incorporating a complete dengue vaccine can change the environment from unpredictability to resilience. It is evident that parenteral fluids, like all therapeutic procedures, have both positive and negative effects and that these effects change depending on the clinical setting. Truly, evidence-based guidelines are being developed for many critical care scenarios; however, these research studies are infrequently conducted in low- and middle-income nations where the illness burden is frequently high and facilities and resources are typically scarce. The multi-organ involvement in dengue shock syndrome necessitates a comprehensive and multidisciplinary approach to patient care.
